# Transmission Optimization Metrics Setup Issues in the Field of Time Constrained Communications

**DOI:** 10.3390/s18093104

**Published:** 2018-09-14

**Authors:** Ondřej Vondrouš, Zbyněk Kocur, Jaromír Hrad

**Affiliations:** Department of Telecommunication Engineering, Faculty of Electrical Engineering, Czech Technical University in Prague, Technická 2, CZ-166 27 Praha 6, Czech Republic; zbynek.kocur@fel.cvut.cz

**Keywords:** Transmission Optimization Metric, protocol optimization, sensor networks, narrow-band networks, inter-packet gaps analysis

## Abstract

This article introduces a new approach in the field of network optimization based on Transmission Optimization Metric (TOM), which is aimed at improving traffic flow continuity and increasing the chances for traffic flow sustainability in a way that helps to minimize inter-packet gaps. The work is mainly focused on harsh transmission conditions in narrow-band networks. Finally, the presented approach has impact on better resource allocation as fewer attempts are necessary for successful completion of a transmission. A significant part of the article deals with parameterization of coefficients used by the TOM optimization method. Examples of analysis for several topologies of narrow-band wireless networks based on CSMA/CA and TDMA protocols are used to demonstrate various issues related to proper setting of parameters. The introduced TOM metric has the potential to become a standard for optimization, for example, in sensor networks that are characterized by the specific nature of data traffic.

## 1. Introduction

The complexity of the design of telecommunication devices and systems grows with demands for improving their parameters and quality. The design of wireless devices using complex modulation and transmission techniques is no longer just the matter of exact mathematical and physical apparatus. In the case of Wireless Sensor Networks (WSN) or Internet of Things (IoT) networks, the problematics is even more complicated because very limited computational and power resources have to be taken into account [1,2]. In particular, efficient design of telecommunication equipment for the respective device/network and its pilot testing require deployment of simulators. A properly designed model and simulation can be used to verify the design, to optimize it, and also to identify possible faults in a real system.

The performing of a simulation itself is not sufficient for obtaining the feedback on the behavior of the communication system–it must be supplemented by a detailed analysis and subsequent evaluation of the data. Then the evaluation results can be used as feedback when optimizing or modifying the simulated communication system.

This article introduces a new Transmission Optimization Metric (TOM) based on inter-packet gaps or packet observation time differences. The main objective of this article is to introduce the TOM metric and its benefits, to show specific setup issues and their impact on the outcomes.

The evaluation system is applied to simulation results of TCP/IP communication protocol running in a Point-to-Multipoint narrowband wireless network based on CSMA/CA and TDMA with channel width up to 25 kHz, 4-CPFSK fixed modulation and 3/4 FEC. The maximum speed achieved on real equipment in point-to-point mode is approximately 15 kbit/s in half-duplex mode. Approximately 7% difference in throughput is assumed in the simulation model of CSMA/CA system (this value is based on previous measurements, the purpose of which was to compare the quality of the simulation model implementation with a real device). The simulation model is implemented in a discrete OMNeT++ [3] simulation environment (version 5.2.1) using the INET [4] extension (version 3.6.3). More detailed information on the implemented model is given in [5].

## 2. State of the Art

The evaluation of communication networks simulations is a very complex task. Methods convenient for network traffic optimization lie in several areas.

At first we can focus on methods based on ITU-T and IETF recommendations. For optimization purposes, the recommendations from the range of ITU-T Y.1500-1599 (Y.1540, Y.1545.1, Y.1560, Y.1563 and Y.1564) [6,7,8,9,10] are most important. As for the IETF, we also found some important recommendations (RFC 2330, RFC 2544, RFC 6349, RFC 7312 and RFC 7799) [11,12,13,14,15]. These represent several decades of network testing and bench-marking, but still, if we are interested in improving data flow continuity rather then overall performance or availability of service, they do not provide the desired support.

In addition to the routine statistical evaluation concerning the frequency of transmitted packets, determining the throughput, delay or loss, several other parameters can be obtained. In the field of communication systems using the TCP/IP family of protocols, we are talking mainly about the fairness analysis of the individual TCP connections by using some of the fairness index options, such as epsilon fairness [16], proportional fairness [17], max-min fairness [18] and Jain’s fairness index [19]. It is quite common that metrics based on Jain’s fairness index (JFI) are used for transport protocol design to ensure specific level of fairness among multiple traffic flows. We can assume that fair resource allocation leads to improved continuity of traffic flows, but demands on fairness in resource allocation can be too strict and unnecessary in specific scenarios.

Another way is to determine the success rate of the individual transmissions. A typical representative is the set of evaluation metrics CER/TER [20]. This metric is based on ratio of unsuccessful/total connections and transmission reflecting communication issues and connection timeouts. This metric is able to partially reflect data flow continuity. It is also possible to increase sensitivity of CER/TER metric to TCP timeout, in limited extent, by decreasing the Endpoint Reuse Interval (ERI) when processing PCAP data with the tcptrace tool [21].

Depending on the particular type of communication used, other parameters can be evaluated, mostly those related to the internal function of the respective communication protocol or the whole system. Most of these parameters require that the evaluation is performed on a captured data block. Continuous (per packet) analysis significantly reduces the accuracy and credibility of the obtained information. This method of analysis uses principles of link parameters acquisition used in communication protocols SCTP [22] and RSTP [23]. The features and mechanisms used in these protocols were an inspiration for developing our own metric: the analysis based on inter-packet gaps (IPGA) measurement, which serves as a basis for the TOM optimization metric.

The monitored parameters (transmission rate, delay and loss rate) in narrow-band wireless networks are very sensitive to the current load of transmission path. Even a small change in the sequence of packets or their holding may significantly influence the transmission parameters or even lead to breakdown of the connection. In broadband networks, this phenomenon is not very noticeable: it occurs mainly due to a sustained load of the transmission channel by a limit data traffic.

The sequence of packets and the delays between their arrivals are affected by several factors. If we ignore intentional use of QoS control mechanisms (Traffic Shaping, Traffic Policing) then another important factor influencing these parameters consists in the used access mechanism of the second-layer (L2) protocols. CSMA-based mechanisms show more efficient bandwidth use, but at higher load (more than 28%) there occurs rapid deterioration of the monitored parameters and more frequent disruption of the established TCP connections [24]. TDMA communication protocols can provide much better transmission parameters, especially for delay-sensitive applications. TOM optimization results can be advantageously used when designing and parameterizing time sessions in the L2 protocols of ISO/OSI RM.

### 2.1. Inter-Packet Gaps Analysis

**Figure 1:**
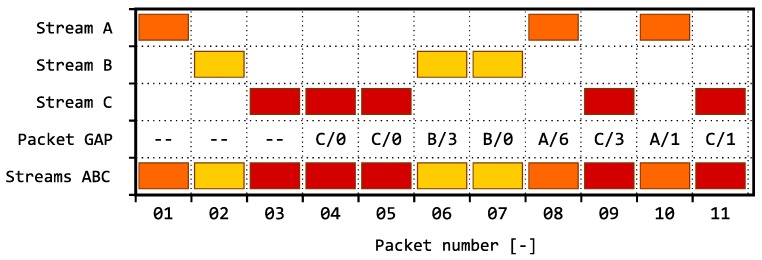
Example of using IPGA: analysis of absolute gap between two consecutive packets (letters indicate the packet origin, and numeric values stand for the number of intermediate packets).

**Figure 2:**
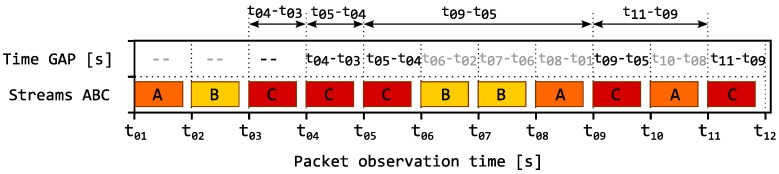
Example of using IPGA: analysis of time difference between two consecutive packets.

We define the term inter-packet gaps analysis (IPGA) as the basic approach to evaluation of gaps qbetween the arrivals of packets within one or several TCP/IP connections. The analysis can be performed for both connection-oriented (TCP) and connectionless (UDP) transmissions. The observed inter-packet gaps metrics can be described in two ways:

- The number of packets passing through an IP connection between two consecutive packets within the monitored IP connection; see illustration of the principle in Figure 1.
- The time between the arrivals of two consecutive packets within a single IP connection; see illustration of the principle in Figure 2.

The inter-packet gap directly affects a variety of transmission path parameters; in the first place, however, the overall delay, including its fluctuations. With connection-oriented protocols (TCP), it influences the transmission rate and round-trip time (RTT). For connectionless protocols (UDP), the arrival times of the individual packets are affected, and thus the quality of service (QoS). In particular, multimedia applications are very sensitive to this fluctuation [25]. This is rather simple approach, but it helps to reveal network communication issues. During our measurements of CSMA/CA scenario we witnessed packet gaps larger than one thousand packets (near the boundary of communication failure due to TCP communication timeout of 1800 s) in uninterrupted communication consisting of 16 parallel streams.

### 2.2. Transmission Optimization Metrics

We developed the new Transmission Optimization Metric (TOM) to evaluate benefits of optimization in narrow-band networks. The TOM metric is not bound to any specific area of network optimization. It can reflect changes made to all layers of TCP/IP communication stack. It relies on the specified inter-packet gap boundary (packet- or time-based), classifying the packets into two groups. The group of packets that exceed the required boundary (f(n) function) is given in relation to all monitored packets as defined by Equation (1), where $F_{num}$ is the number of flows, $N_{f}$ is the number of packets in the observed flow, *N* represents the number of all packets in all flows, and *k* is the number of processed packets.

(1) $$\mathrm{TOM}=1-\sum_{id=1}^{F_{num}}\frac{\sum_{k=1}^{N_{f}}f\left(k\right)}{N}$$

TOM metric outputs are in range $\langle 0,1\rangle$. This range is appropriate for comparison with other standardized evaluation metrics from the FI (Fairness Index) family.

Large values close to 1 indicate that in the data stream under evaluation there are only minimum of packet gaps which exceed the demanded limit. On the contrary, low values indicate that the inter-packet gaps exceed the demanded limit at a higher rate.

Similarly to the IPGA, two approaches to evaluation of inter-packet gaps can be used in TOM. When using the TOM on inter-packet gaps, the $f_{g}\left(n\right)$ function is defined as follows, where G represents the boundary defined through the number of intervening packets from other streams:

$$f_{g}\left(n\right)=\left\{\begin{array}{cc} 1, & \mathrm{if}\;packet\;gap>G\\ 0, & \mathrm{else} \end{array}\right.$$

When using the TOM on packet time differences, the $f_{t}\left(n\right)$ function is defined as follows, where *T* represents the boundary defined as a time difference between packets in the specific stream:

$$f_{t}\left(n\right)=\left\{\begin{array}{cc} 1, & \mathrm{if}\;time\;difference>T\\ 0, & \mathrm{else} \end{array}\right.$$

The main idea of this approach is that it is not necessary to deliver packets in the shortest possible time, but it is more important to maintain a specific level of flow continuity. Especially in TCP communication this leads to improved sustainability as it relies on limiting the number of packet gaps (time-based in this case) exceeding the allowed boundary.

## 3. Optimization Methodology

The TOM optimization methodology is based on the classification of inter-packet gaps in the measured or simulated network and their subsequent evaluation according to the given criteria that have to be defined with regard to the requirement to improve sustainability or to respect the specific request flow continuity for each connection during transmission in a narrow-band network. The transmission system is subsequently optimized in order to avoid the occurrence of L2 to L4 protocol timeouts, or to adhere to strictly given requirements for application-level message delivery (e.g., in SCADA systems).

**Figure 3:**
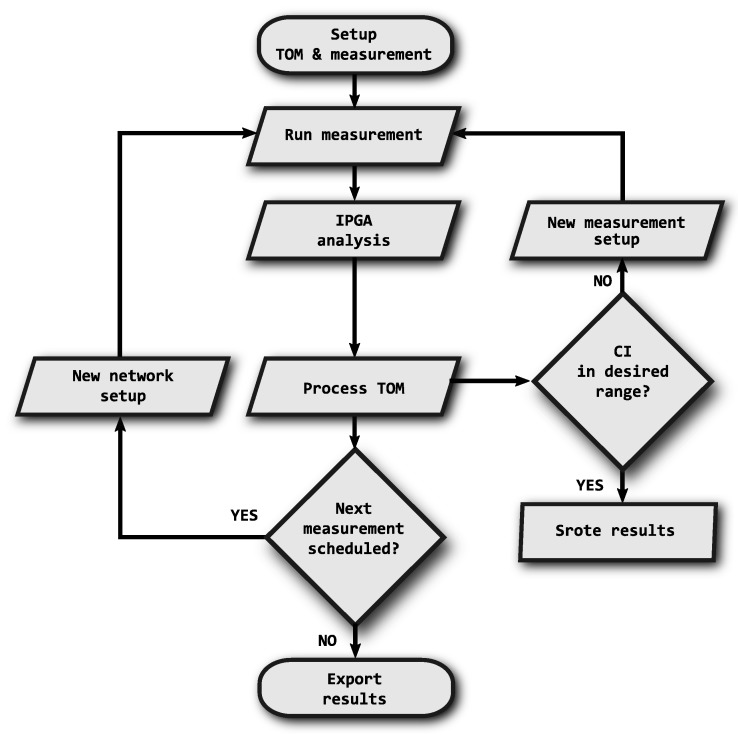
Flowchart of the analysis and evaluation process according to IPGA and TOM.

The fundamental optimization algorithm is shown in Figure 3. Each step corresponds to the description provided in the following list.

1. The first step is to set up measurement scenarios that define the extent of the performed analysis in relation to the required reliability of the results. Within each scenario, the critical parameters are the duration of measurement, the number of iterations and the default inter-packet gap for the TOM metric evaluation. It is also possible to set the desired size of confidence interval (CI).
2. Then follows the actual measurement according to the defined scenarios and their parameters. When multiple repetitions are scheduled, change of random number generator (RNG) seed is performed.
3. The IPGA analysis is performed with the obtained data, providing inter-packet gaps as its output.
4. Calculation according to the TOM metric is applied to the inter-packet gaps.
5. From the obtained results, the confidence interval [26] is calculated and compared to the required value that is set at the beginning of the measurement. If the confidence interval is too large, a correction must be made by adjusting the duration of the measurement or by increasing the number of repetitions; then the measurement is repeated with the new parameters.
6. If there are any further measurements not yet performed, they are carried out according to items 2–5 with a new network and measurement setup (including RNG seed setup).
7. At the end, the results are exported and evaluated.

## 4. TOM Verification

The presented metric reflects the ratio of packets exceeding the defined boundary. That results in outcomes which only depend on the boundary setup. In this case we have a metric that is able to help in optimizing the traffic continuity by minimizing the maximum packet delays.

**Figure 4:**
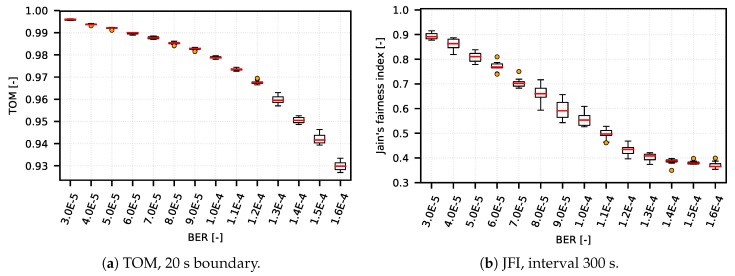
Scenario DumbBell, TOM and JFI comparison.

**Figure 5:**
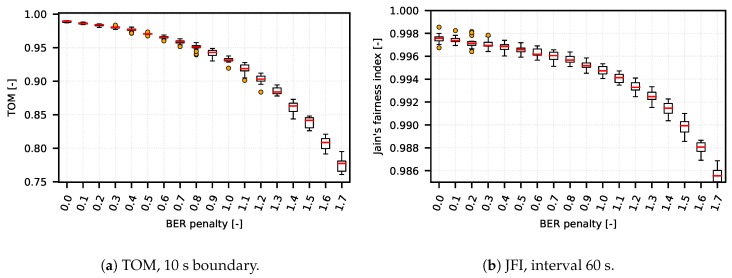
Scenario TDMA, TOM and JFI comparison.

We verified (performing from 20 to 30 measurements with the same settings and different RNG seed setu—the results are represented by boxplot diagrams) that the TOM metric is capable of reflecting network conditions in a wide range of network setups (from simple artificial DumbBell scenarios to complex CSMA/CA and TDMA systems based on real deployment). On one hand, the TOM metric performs as expected under variety of network conditions; on the other hand with specific setup, when compared to a metric based on JFI, the results show that the TOM metric is capable of delivering almost the same results as the metric based on JFI, as shown in Figure 4a,b and Figure 5a,b.

When compared to JFI-based metrics, the main difference consists in the fact that TOM metric depends only on packets exceeding the defined boundary, and the distribution of packets among streams is not important. With TOM, the network is optimized in a way that minimizes the number up packet gaps exceeding the defined boundary. This has direct impact on traffic flow continuity, resulting in increased sustainability of communication.

## 5. Issues Related to TOM Boundaries Selection

The TOM outputs are strongly dependent on the setting of the inter-packet gaps boundary in both the packet and time domains–see the decision functions $f_{g}\left(n\right)$ and $f_{t}\left(n\right)$. If we focus purely on maximizing the connection sustainability, it may not be a simple task to find the correct boundaries for parameterization of the TOM metric.

If the information about lower-layer protocols and their configurations is available, it is possible to use it for parameterization. Typically, the information about settings of different protocol timers can be used for setup and optimization. In the case of communication optimization in narrow-band networks, the boundary values of parameters in the time domain can reach hundreds of seconds, but they should be less than the total expiration time for the TCP connection.

The situation is even more difficult when we need to push optimization further to improve also communication delays of the optimized traffic flows. Given the much stricter requirement for setting the boundary values (compared to the requirement for connection sustainability), it is necessary to perform the configuration much more carefully.

The real problem, however, occurs when the TOM metric should be used purely to minimize the inter-packet gaps. If the only criterion is to reach the minimum, the question arises about the initial setting of the metric. During the basic testing we chose a procedure that relies on one set of measurements, which is considered the initial state; then we compare it to the results of measurements with modified parameters and decide whether or not the observed criterion has been improved. And right here we are encountering the problem of how to set the initial TOM boundary values so that the pros and cons of each setting modification can be evaluated during the whole measurement with the different configurations.

**Figure 6:**
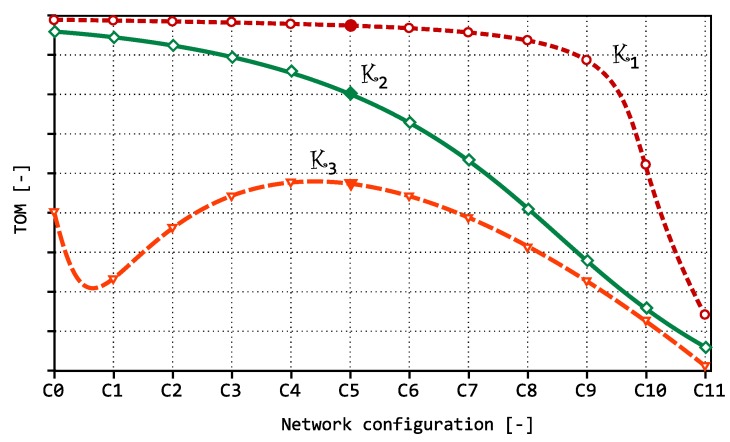
Optimization curves.

Figure 6 shows a simplified view of possible optimization curves. If we assume that the differences in the settings are not significant (that is, if a small change of the settings does not assume a major change in the distribution and length of inter-packet gaps), the intermediate values can be interpolated with a curve.

- The curve $K_{1}$ shows a situation when, within a defined criterion, it is evaluated that the settings from the initial part of the graph should be used as the optimum; however, the slope of the curve is minimal here, which may cause problems in distinguishing the benefits of the individual optimizations. In the remaining part of the chart, there is usually a sufficient slope, but the area is not interesting from the optimization point of view (TOM value decreases, and there is no maximum nor a value close to it).
- The curve $K_{2}$ represents the desired optimal shape, which should allow evaluation of how the individual optimizations affect the monitored network traffic. The monotone waveform can be monitored mostly where the optimal state in a simple form depends on the value of the optimized criterion (for example, improving network throughput due to decreasing latency).
- The curve $K_{3}$ represents another appropriate shape, but the optimum is not located at any of the graph ends. This occurs when there is a more complicated relationship between inter-packet gaps and optimization parameters. Here the actual strength of the TOM optimization metrics is demonstrated, because in specific cases it can detect optimal settings that may not match the original assumption. Generally, it is assumed that the transmission conditions—and hence the inter-packet gaps—are proportional to error rate during data transmission; but this is not always true, as shown below.

The shape of the curve $K_{1}$ shows that for roughly two thirds of the values, it will be difficult to compare them and, moreover, in the area where the optimum is located, according to the chosen optimization criterion. This problem could be solved by lowering the boundary gap value when comparing the inter-packet gaps within the TOM metric setting. However, as will be shown below, in such case the optimum is often shifted to another area of the graph, and therefore another setting emerges as the optimal one.

The shape of curves $K_{2}$ and $K_{3}$ can be considered optimal for several reasons. The curve and its slope must have such shape that the neighboring values can be clearly distinguished from each other. This means that it must be possible to identify situations in which the TOM metric reaches or approaches the highest value. It is also required that the shape of the curve corresponds to the trend of the optimized values according to the chosen optimization criterion. That is, we assume a significant change in shape and waveform of the curve with deflection from the optimum in response to a change of the input conditions that ultimately results in increased values of inter-packet gaps or in a higher number of packets with inter-packet gaps larger than the selected boundary value for TOM metric evaluation.

## 6. Demonstrative Measurements

The actual use and verification of the proposed TOM methodology is demonstrated in several real examples (scenarios). The samples used were selected with respect to the specific features to reveal issues related to TOM boundary settings. We prepared several scenarios such as simple DumbBell topology with shared channel and FIFO queue, CSMA/CA and TDMA transmission systems to outline the mentioned issues. As simulation environment we used OMNeT++ with INET framework.

The aim of the following examples is to demonstrate the way how the TOM metric can be used, but mainly to illustrate the outcomes of specific TOM boundary value setting.

### 6.1. Scenario: DumbBell Network Using Shared FIFO Queue

In this scenario, a simple DumbBell [27] communication network with 12 client stations is simulated. The client stations download data from a server using the basic TCP protocol implementation according to RFC 793 [28,29]. The communication originates in the client stations where the individual TCP connections are initialized in a short time window, and the main transmission of data from the server to the clients runs simultaneously. The simulation of a narrow-band network physical layer was very simplified. The shared transmission medium was implemented using an ordinary FIFO queue, ensuring its uniform filling from all contributing streams. For this shared medium, throughput was limited to 19 kbit/s and delay was set to 50 ms. For the sake of clarity and especially predictability of the simulated network behavior, the error rate of the shared medium was chosen as a variable parameter. Within the performed simulations, it ranged from 3 × $10^{-5}$ to 1.6 × $10^{-4}$ to create conditions similar to wireless narrow-band networks. The values are inspired by the findings of the works mentioned in [30,31].

**Figure 7:**
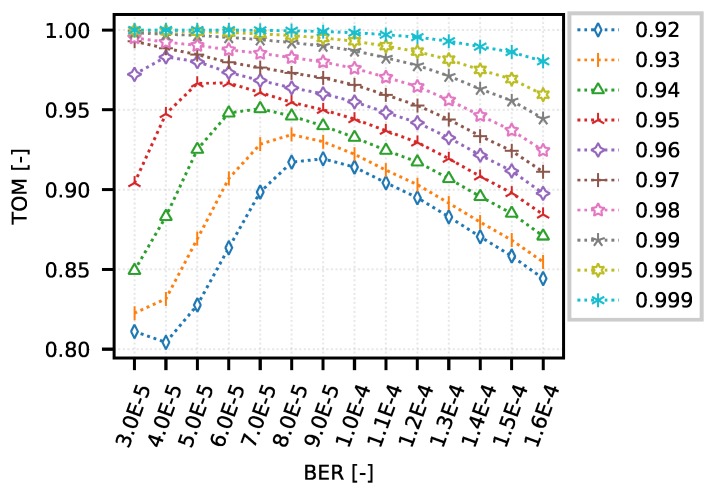
DumbBell—example of optimization curves (mean quantiles 0.92–0.999 of BER 9 × $10^{-5}$ configurations).

Figure 7 shows the optimization curves if the input value used to set the TOM metric boundary corresponds to the selected quantile (obtained from the IPGA, where measurement at BER 9 × $10^{-5}$ was taken as a reference). An overview of the quantiles and their corresponding values are given in Table 1.

If we take a closer look at the values of the TOM metric, which correspond to the values of the 0.95 and 0.99 quantiles, it is clear from the graph that if we choose a boundary gap of approximately 5 s (0.95 quantile), then it appears that this criterion best meets a network where the error rate is approximately 5 × $10^{-5}$–6 × $10^{-5}$. This is probably due to the fact that the error rate causes a reaction from the TCP control algorithms, and thus it is regulated to a level where individual data streams are more equitably assigned the available network resources. In the case of network optimization for a longer inter-packet gap, i.e., approximately 39 s (0.99 quantile), it seems advantageous to keep the error rate as low as possible.

### 6.2. Scenario: Narrow-Band Network Using CSMA

The scenario with a narrow-band network using CSMA/CA protocol is based on a real implementation of this protocol into the simulation environment. Configuration of the simulated network is derived from a real-life configuration of narrow-band modems operating in the frequency band between 140 and 960 MHz using channel width of 25 kHz (or alternatively 12.5 kHz and 6.25 kHz). The CSMA/CA protocol is very efficient by allocating of network resources to individual communicating nodes, but also highly susceptible to congestion, which consequently leads to a significant impact on or even the breakdown of the active connections. This is a field for further optimization.

**Figure 8:**
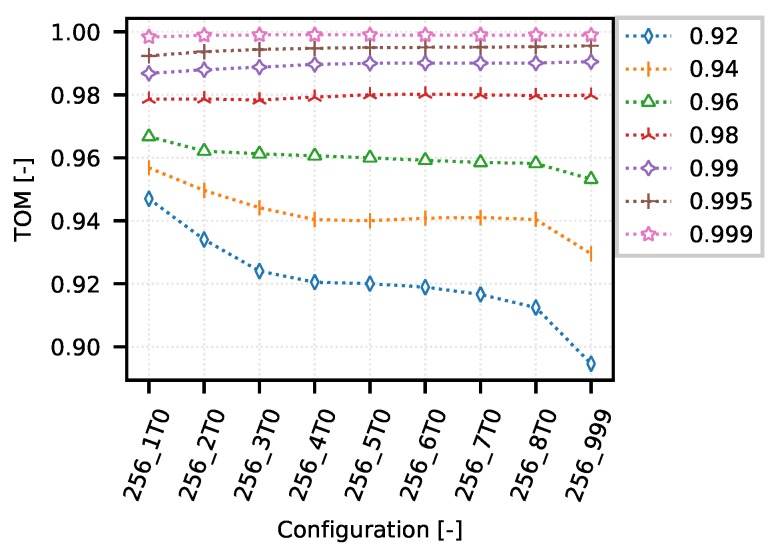
CSMA/CA—example of optimization curves (mean quantiles 0.92–0.999 of 256_5T0 configurations).

On one hand, the following example demonstrates how the TOM metric can be used to optimize narrow-band communication systems. On the other hand, it shows typical obstacles in TOM boundaries selection. The Figure 8 shows several TOM metric cases that reflect different settings of the optimization criterion value corresponding to the specific quantile of the analyzed values according to the IPGA. An overview of the quantiles and their corresponding values is given in Table 2. The configurations on presented graph reflects different settings of queuing timeouts (from 1 s to 8 s, and also unlimited labeled as “_999”) with limitation of TCP MSS to 256 B.

As can be seen from Figure 8, the simulations of a CSMA/CA system provide much less space for comparing the TOM metric values when the optimization of communication is performed for values corresponding to 0.98 quantile and higher. This is mainly due to the nature of the considered network traffic and measurement conditions.

**Figure 9:**
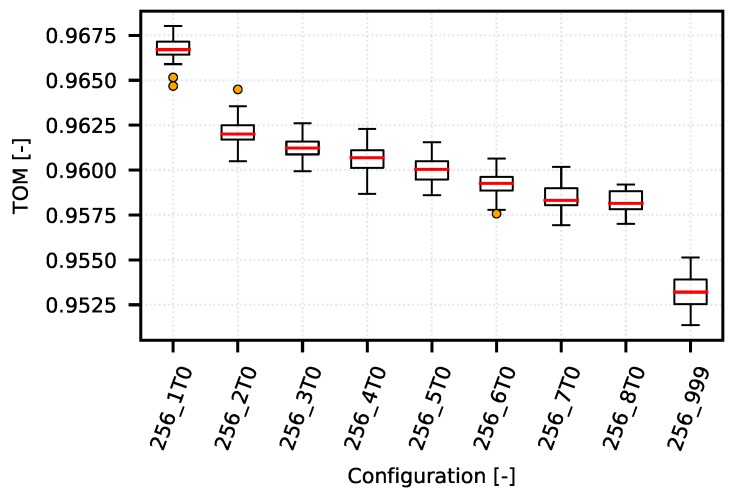
CSMA/CA—example of optimization curve (mean quantile 0.96 of 256_5T0 configurations).

**Figure 10:**
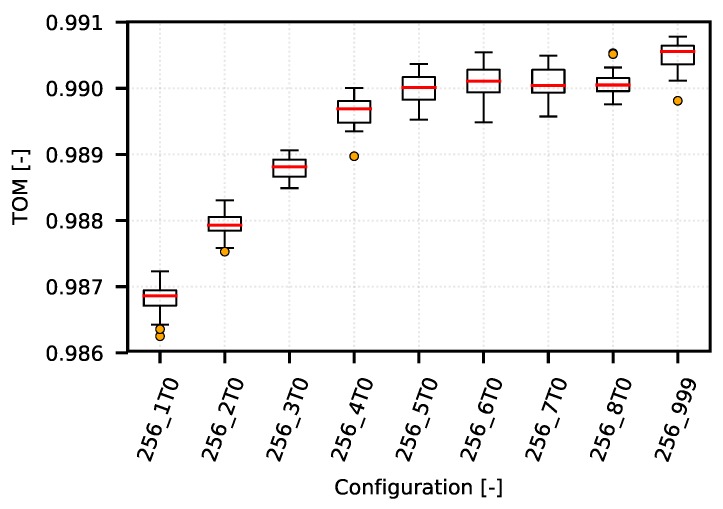
CSMA/CA—example of optimization curve (mean quantile 0.99 of 256_5T0 configurations).

**Figure 11:**
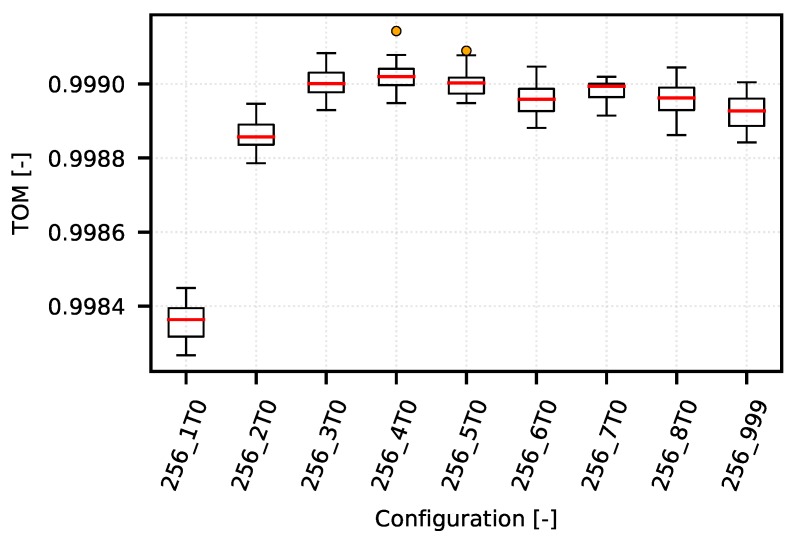
CSMA/CA—example of optimization curve (mean quantile 0.999 of 256_5T0 configurations).

However, as shown in a detailed view of the individual optimization curves for quantiles 0.96 (Figure 9), 0.99 (Figure 10) and 0.999 (Figure 11), the TOM-based analysis can be used also in these cases, although it is necessary to consider that deciding about the optimal setting may be problematic. This is caused by too little separation of the individual TOM metric values. In this case, it is necessary to apply statistical analysis methods and to determine the reliability of the given results.

As for the TOM metric values for the 0.96 quantile (Figure 9), the optimum lies within the configuration area “256_1T0”. In this case, the network is optimized to maintain the time differences below the level of approximately 15 s.

For the 0.99 quantile (Figure 10), the optimum lies on the opposite side of the graph in the configuration area “256_T999”. In this case, the use of the 0.99 quantile corresponds to the optimization boundary of approximately 40 s.

For the 0.999 quantile (Figure 11), the optimum lies in the central area of the graph. In this particular case, the network is optimized for a request to maintain connection rather than for a specific inter-packet gap. The optimization boundary is set to more than 200 s.

### 6.3. Scenario: Narrow-Band Network Using TDMA

The scenario uses the TDMA access method for communication among devices in a narrow-band network. Round-robin algorithm was used for resources allocation to the communication parties. For the sake of simplicity, the network structure was chosen similarly as in the DumbBell scenario, including the maximum throughput of 19 kbit/s. The delay is given by the used L2 algorithm and by the number of connected clients. The network setup allows direct comparison of simulation results between DumbBell and TDMA scenarios. The simulation was also parameterized using physical channel error rate. As each node connection was parametrized via different level of BER, we introduced special parameter called BER penalty in the measurements, being used to increase the global probability of bit errors in whole network (with BER penalty 1.2 and the original BER of 1 × $10^{-5}$, the new calculated BER becomes 1 × $10^{-3.8}$).

When a TDMA-based protocol is used, the situation is quite different. Network resources are allocated according to a scheduler code, and the probability of communication failure due to timer expiration is minimal. In the case of deterministic access protocols, the use of the TOM metric can be expected rather to optimize the connection in such a way that the strict requirements for transmission continuity are met. From the performed measurements and analyses it is possible to imply that there exists a wide range of values for the selection of TOM metric boundary gaps. However, the overall interval, in which the inter-packet gap values corresponding to the selected quantile lie, is very small, due to the very strict allocation of resources by the round-robin algorithm within the testing scenario. An overview of the quantiles and their corresponding values are given in Table 3.

**Figure 12:**
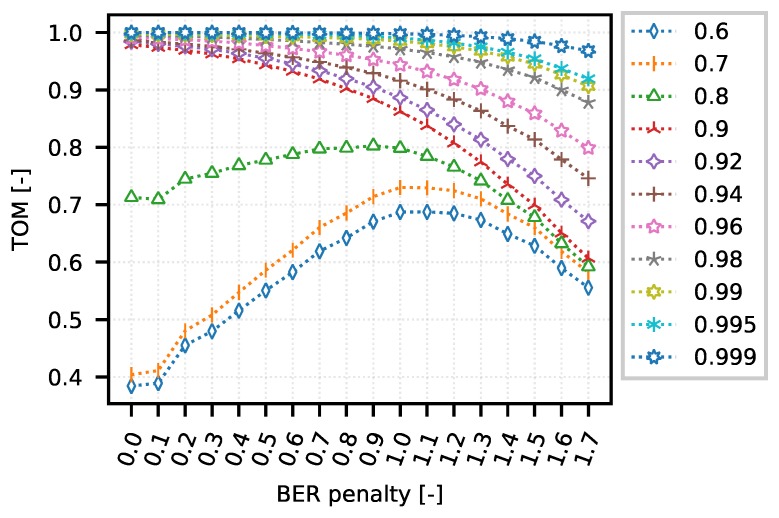
TDMA—example of optimization curves (mean quantiles 0.6–0.999 of BER penalty 0.8 configurations).

Figure 12 shows a set of curves for quantiles 0.6 and higher. In contrast to the above-mentioned technologies, the differences between the corresponding values in the range of 0.6 to 0.9 are minimal. However, this does not alter the fact that there occurs a radical change of the monitored parameters right in such a narrow range of values. It turns out that for the values of setting the boundary gap for the TOM metric corresponding to quantile 0.9 and above, the decreasing error rate has a major impact on reducing the inter-packet gaps. Conversely, for values corresponding to quantiles below 0.9 (in the range of 0.5 to 0.9), it can be observed that a certain increased error rate positively influences the reduction of inter-packet gaps, or the number of packets that exceed the defined boundary value. This corresponds to the results of previous measurements. The discussed area lies around the value of 1.0 (BER penalty).

## 7. Conclusions

In the present article, we have briefly introduced the IPGA method of inter-packet gaps analysis and the TOM metric as a contribution to improving or even replacing the existing metrics based on JFI, especially for such applications that prefer optimization of communication delays to absolute fairness of resources allocation. The main purpose of the article is to demonstrate the issues of TOM boundary selection with respect to improving the packet flow continuity and increasing the probability of connection sustainability. This is achieved by reducing the gaps between packets within individual data streams.

The demonstration is based on verifying the behavior of optimization procedures in several scenarios of wireless narrow-band networks operated under different L2 protocols. The used simulation scenarios are based on artificial implementation (DumbBell scenario) and real implementations of these communication systems and industrial network applications (CSMA/CA and TDMA scenarios).

The performed analyses show the contribution of the TOM-based methodology for improving the properties of optimized data streams in narrow-band communication networks. The analyses also revealed one of the major disadvantages of these optimization procedures, which is a relatively difficult parameterization.

The results show that if we push hard on lowering the TOM boundary, then the TOM maximum shifts to different areas of the presented graphs; that means we optimize the continuity of data stream at the expense of increased error rate. This occurs in situation when we encounter packet gaps caused by transmission control mechanisms. Therefore we have to be aware of such issues and set the optimization boundaries carefully.

Further research and development will now be undertaken in the direction of automation of boundaries selection. Eventually we want to obtain an analyzing system based on the TOM methodology, the parameterization and operation of which would be fully automatic; this will open new deployment capabilities in the form of real-time analysis performed directly in the respective communication devices.

## Figures and Tables

**Table 1:** DumbBell quantiles (mean of BER 9 × $10^{-5}$ configurations).

Quantile	0.92	0.93	0.94	0.95	0.96	0.97	0.98	0.99	0.995	0.999
gap [s]	1.66	2.28	3.36	4.79	6.86	10.16	16.86	38.97	91.48	239.78

**Table 2:** CSMA quantiles (mean of 256_5T0 configurations).

Quantile	0.92	0.94	0.96	0.98	0.99	0.995	0.999
gap [s]	9.86	11.9	15.03	23.89	40.87	80.01	247.08

**Table 3:** TDMA quantiles (mean of BER penalty 0.8 configurations).

Quantile	0.6	0.7	0.8	0.9	0.92	0.94	0.96	0.98	0.99	0.995	0.999
gap [s]	8.39	8.57	8.60	8.67	9.26	9.78	10.19	11.01	11.38	11.68	13.19

## Abbreviations

The following abbreviations are used in this manuscript:

BER	Bit Error Rate
CER/TER	Connection Error Rate/Transmission Error Rate
CPFSK	Continuous-Phase Frequency-Shift Keying
CSMA/CA	Carrier Sense Multiple Access with Collision Avoidance
ERI	Endpoint Reuse Interval
FEC	Forward Error Correction
FI	Fairness Index
FIFO	First In First Out
IPGA	Inter-Packet Gaps Analysis
IETF	Internet Engineering Task Force
ISO/OSI	International Organization for Standardization/Open Systems Interconnection
ITU	International Telecommunication Union
JFI	Jain’s Fairness Index
L2	OSI Layer 2
L4	OSI Layer 4
MSS	Maximum Segment Size (TCP)
PCAP	Packet CAPture
QoS	Quality of Service
RFC	Request for Comments
RNG	Random Number Generator
RTT	Round Trip Time
SCADA	Supervisory Control And Data Acquisition
TCP/IP	Transmission Control Protocol/Internet Protocol
TDMA	Time Division Multiple Access
TOM	Transmission Optimization Metrics
UDP	User Datagram Protocol
WSN	Wireless Sensor Networks
